# Carotenoid-to-(bacterio)chlorophyll energy transfer in LH2 antenna complexes from *Rba. sphaeroides* reconstituted with non-native (bacterio)chlorophylls

**DOI:** 10.1007/s11120-019-00661-6

**Published:** 2019-07-26

**Authors:** Dariusz M. Niedzwiedzki, David J. K. Swainsbury, C. Neil Hunter

**Affiliations:** 1 Center for Solar Energy and Energy Storage, Washington University, St. Louis, MO 63130 USA; 2 Department of Energy, Environmental & Chemical Engineering, Washington University, St. Louis, MO 63130 USA; 3 Department of Molecular Biology and Biotechnology, University of Sheffield, Sheffield, S10 2TN UK

**Keywords:** Carotenoids, Spheroidene, Light harvesting, Transient absorption, Purple bacteria, Light harvesting complex 2

## Abstract

Six variants of the LH2 antenna complex from *Rba. sphaeroides*, comprising the native B800-B850, B800-free LH2 (B850) and four LH2s with various (bacterio)chlorophylls reconstituted into the B800 site, have been investigated with static and time-resolved optical spectroscopies at room temperature and at 77 K. The study particularly focused on how reconstitution of a non-native (bacterio)chlorophylls affects excitation energy transfer between the naturally bound carotenoid spheroidene and artificially substituted pigments in the B800 site. Results demonstrate there is no apparent trend in the overall energy transfer rate from spheroidene to B850 bacteriochlorophyll *a*; however, a trend in energy transfer rate from the spheroidene S_1_ state to *Q*_*y*_ of the B800 (bacterio)chlorophylls is noticeable. These outcomes were applied to test the validity of previously proposed energy values of the spheroidene S_1_ state, supporting a value in the vicinity of 13,400 cm^−1^ (746 nm).

**Electronic supplementary material:**

The online version of this article (10.1007/s11120-019-00661-6) contains supplementary material, which is available to authorized users.

## Introduction

Carotenoids are abundant natural pigments synthesized by higher plants, algae, and bacteria. These pigments are broadly utilized in photosynthesis in which they simultaneously play several roles. In various types of antenna complexes and photosystems, they supplement light harvesting of (bacterio)chlorophylls ((B)Chls) by enhancing absorption in the 400–500 nm range. Simultaneously in many complexes carotenoids also have photoprotective functions. Typically carotenoids quench potentially harmful triplet states of (B)Chls (Demmig-Adams and Adams 1993; Takaichi 1999) and in some cases (B)Chl excited singlet states (Dilbeck et al. 2016; Niedzwiedzki et al. 2016b; Staleva et al. 2015). In addition carotenoids are broadly used by various autotrophic and heterotrophic organisms for coloration (Britton et al. 1995). Most carotenoids are yellow or orange, which results from an electronic absorption in the spectral range between 400 and 550 nm but some rare examples of blue carotenoids with absorption expanded to 650 nm are also known (Polivka et al. 2010). The absorption band that defines carotenoids visible color is associated with the S_0_ → S_2_ electronic transition of the linear carbon–carbon double bond conjugation that is a characteristic structural motif of all carotenoids. This motif means that the spectroscopic properties of carotenoids are idealized by complementary linear polyenes. Polyenes are assigned to a *C*_2*h*_ point group in which the symmetry of the electronic distribution will classify electronic states as rotationally symmetric (A) or asymmetric (B). Electronic transitions between those states are limited to those that change state inversion symmetry (g ↔ u) and Pariser alternancy symmetry (pseudoparity) (+ ↔ −). Consequently for the idealized polyenes (and ultimately for carotenoids) the first possible, S_0_ (1^1^A_g_^−^) → S_1_ (2^1^A_g_^−^), electronic transition would be strictly forbidden due to the same (A_g_) symmetry and (−) pseudoparity of both electronic states (Christensen 1999; Hudson and Kohler 1974; Schulten and Karplus 1972). However, in a recent review on theoretical modeling of electronic excitations in polyenes, the authors emphasized that pseudoparity labels used to explain allowedness of electronic transitions for real polyenes and carotenoids should be treated as an exclusive feature of computational models and not be applied to real, typically asymmetric systems (Schmidt and Tavan 2012). That conclusion implies that for real polyenes and carotenoids transitions like S_0_ → S_1_ would be in principle allowed, although they have not yet been observed in absorption spectra of almost all of the hundreds of carotenoids and polyenes currently known (Britton et al. 2004). Very recently two carotenoids, synthetic deoxyperidinin (Greco et al. 2016) and geometric central-*cis* isomer of naturally occurring peridinin (Niedzwiedzki and Blankenship 2018), were proposed to reveal S_0_ → S_1_ in their absorption profiles. Presence of S_0_ → S_1_ absorption band was also proposed in cryogenic absorption spectra of short peridinin analogues (Niedzwiedzki et al. 2013). In order to explain complete absence of features associated with S_0_ → S_1_ transition in almost all other carotenoids, it was suggested that a displacement of both S_0_ and S_1_ electronic states plays a crucial role (Fiedor et al. 2016). It was assumed that both states are so significantly displaced that direct access from the ground state to S_1_ would require molecular deformation that simply cannot be achieved, and the transition is essentially silent due to lack of overlaps in Franck–Condon factors (Fiedor et al. 2016). These show that the understanding of excited state properties of carotenoids are far from complete (Hashimoto et al. 2018).

Numerous carotenoids are found in the light harvesting 2 (LH2) complexes produced by purple phototrophic bacteria. LH2 is a transmembrane pigment–protein complex playing the role of the peripheral light harvesting antenna expanding both absorption cross section and pigment density of the photosynthetic apparatus. It absorbs light and efficiently transfers excitation energy to the light harvesting complex 1 (LH1), which is bound to the reaction center where conversion of electronic excitation energy to chemical energy occurs (Cartron et al. 2014; Qian et al. 2013). The LH2 complex is a circular structure built from multiple heterodimers of α and β polypeptides. Each αβ heterodimer houses three BChls *a* and one carotenoid (which varies depending on bacterial species). Spectrally, LH2 complexes contain two distinct populations of BChl *a* designated B800 and B850, which is a consequence of their organization within the LH2 protein scaffold. The names of the BChl *a* populations are derived from the wavelength positions at which their *Q*_*y*_ absorption bands appear. The B800 ring is a set of weakly coupled monomeric BChl *a* molecules with their macrocycles oriented parallel to the plane of photosynthetic membrane, located between adjacent pairs of αβ polypeptides. The B850 ring is an array of strongly coupled BChl *a* dimers located within each αβ subunit with macrocycles oriented perpendicular to the membrane plane (McDermott et al. 1995; Papiz et al. 2003; Prince et al. 1997). Two high-resolution X-ray structures of LH2 are available from two non-sulfur purple bacteria, *Rhodopseudomonas acidophila* strain 10050 and *Phaeospirillum molischianum* (Koepke et al. 1996; McDermott et al. 1995; Papiz et al. 2003; Prince et al. 1997) as well as low-resolution projection structures of LH2 from *Rba. sphaeroides* (Walz et al. 1998) and *Allochromatium vinosum*. These structures demonstrate that LH2 adopts circular form and has from 8 to 13 αβ subunits, depending on species (Kereiche et al. 2008; Koepke et al. 1996; McDermott et al. 1995; Papiz et al. 2003; Prince et al. 1997).

Spheroidene (Sphe) is an open chain carotenoid produced as the major carotenoid in many species of purple phototrophic bacteria including the model organism *Rba. sphaeroides* (Takaichi 1999). *Rba. sphaeroides* LH2 complexes containing Sphe have been extensively studied with various static and time-resolved spectroscopic techniques (Angerhofer et al. 1995; Cong et al. 2008; Dilbeck et al. 2016; Fidler et al. 2013; Jimenez et al. 1996; Ostroumov et al. 2013; Polivka et al. 2002; Trinkunas et al. 2001). Sphe has nine conjugated carbon–carbon bonds in its backbone, a property that determines appearance of its absorption band at ~ 485 nm (0–0 vibronic peak) in low polarizable solvents like ethanol (Britton et al. 2004). Within the LH2 protein environment the 0–0 vibronic peak shifts to ~ 510 nm. Excited state properties of Sphe in various solvents have previously been studied at room temperature (RT) as well at 77 K. The lifetime of its S_1_ excited electronic singlet state is in the 7–9 ps range at RT and slows to ~ 12 ps at 77 K (Frank et al. 1997, 2000; Niedzwiedzki et al. 2007, 2009; Polivka et al. 1999, 2001, 2005; Rondonuwu et al. 2003; Zhang et al. 2000). For Sphe bound to the LH2 complex, the S_1_ excited state lifetime is substantially shorter (~ 1 ps) due to the fact that it efficiently transfers excitation energy to both B800 and B850 BChls (Cong et al. 2008; Polivka et al. 2007; Rondonuwu et al. 2004). As Sphe does not show a detectable absorption to the S_1_ electronic state, the exact value of the S_1_ state energy is still debatable. The S_1_ state energy level was previously investigated by spectroscopic methods including fluorescence emission (Fujii et al. 1998), resonance Raman scattering (Sashima et al. 1998) and S_1_ → S_2_ transient absorption (Niedzwiedzki et al. 2009; Polivka et al. 2001). The published S_1_ state energy levels were sensitive to the particular experimental techniques, yielding values spanning almost a 1000 cm^−1^ range (13,400–14,200 cm^−1^; 746–704 nm) in various solvents. Based on S_1_ → S_2_ TA spectra of LH2 containing Sphe, it was estimated that the S_1_ state energy of protein-bound Sphe is 13,400 cm^−1^ (Polivka et al. 2002) suggesting that the protein environment has little influence on the state energy. However, this value has not been confirmed with other techniques or by other research groups.

LH2 complexes from *Rba. sphaeroides* and *Rhodoblastus acidophilus* with alternative B800 pigments have previously been used to study energy transfer between the B800 and B850 (B)Chls and B850 LH2 complexes have previously been used to elucidate the role of B800 BChl *a* in carotenoid → B850 energy transfer (Bandilla et al. 1998; Herek et al. 2000; Linnanto and Korppi-Tommola 2002; Macpherson et al. 2001; Swainsbury et al. 2019). The primary focus of those studies was to elaborate excitation energy transfer between those (non-)native (B)Chls and B850 BChl *a* and to test the viability of in vivo pigment replacement to expand spectral coverage via protein engineering. However, a set of these LH2 complexes is also an excellent platform to study how energetic variation of the “B800” *Q*_*y*_ band affects the ability of Sphe to transfer excitation from its S_1_ state to the (B)Chl within the B800 site, which is presented in this work. We demonstrate that the rate of energy transfer from the Sphe S_1_ state to “B800” is sensitive to the position of the (B)Chl *Q*_*y*_ band, providing another indirect method to probe the energy level of S_1_ state of Sphe within LH2. The results support an S_1_ state energy of LH2-embeded Sphe in the vicinity of 13,400 cm^−1^ (746 nm).

## Materials and methods

### Bacterial growth and LH2 purification and reconstitution with non-native (B)Chls

*Rba. sphaeroides* cells lacking the CrtA gene [reported previously in (Chi et al. 2014)] were grown under 30 μmol photons s^−1^ m^−2^ illumination from OSRAM classic 116 W halogen bulbs in M22+ medium (Hunter and Turner 1988) supplemented with 0.1% w/v casamino acids. B800–B850 LH2 was purified by ion-exchange chromatography followed by size-exclusion as previously described (Swainsbury et al. 2019). To generate a set of LH2 complexes with alternative (B)Chls bound to the B800 site, purified LH2 was exchanged into pH 5.0 buffer containing lithium dodecyl sulfate (LDS). LDS selectively removes the B800 BChl *a* resulting in a loss of its characteristic 800 nm peak generating B850 LH2 named for its sole 850 nm band in the near-infra red region (Clayton and Clayton 1981; Fraser et al. 1999; Kramer et al. 1984; Robert and Frank 1988). Subsequently, B850 LH2 was incubated in excess of alternative (B)Chls (Chl *a*, Chl *d*, acChl *a* and BChl *b*) that readily bind to the vacant B800 site yielding complexes with new spectral features. For a detailed protocol see (Swainsbury et al. 2019).

### Spectroscopic methods

Room temperature (RT) and 77 K steady-state absorption spectra were recorded using a UV-1800 spectrophotometer (Shimadzu, Japan). Fluorescence emission and excitation spectra of LH2 complexes were recorded at RT using an RF-6000 spectrofluorometer (Shimadzu, Japan). Fluorescence emission spectra were measured following excitation of the (0–0) vibronic peak of the carotenoid (Sphe) band at 510 nm with excitation and emission bandwidths of 5 nm, and with a 570-nm long pass glass filter at the detector entrance. Fluorescence excitation spectra were collected monitoring emission at 850 nm with excitation and emission bandwidths of 5 nm, with a 665-nm long pass glass filter at the detector entrance. Cryogenic measurements at 77 K were taken using a VNF-100 liquid nitrogen cryostat (Janis, USA). The samples were resuspended in 60/40 glycerol/buffer (v/v) mixture placed in 1-cm polymethyl methacrylate cuvettes and slowly frozen in nitrogen vapor.

Transient absorption (TA) experiments were carried out using Helios, a femtosecond time-resolved pump-probe absorption spectrometer (UltrafastSystems LLC, USA) coupled to a Spectra-Physics femtosecond laser system described in detail previously (Niedzwiedzki et al. 2016b). All LH2 complexes were excited at 510 nm corresponding to (0–0) vibronic peak of Sphe absorption band. The energy of the excitation beam, focused on the sample in a spot approximately 1 mm diameter, was 200 nJ corresponding to 7 × 10^13^ photons cm^−2^ per pulse.

### Data processing and global analysis

Prior to analysis, TA datasets were corrected for temporal dispersion using Surface Xplorer 4.0 (UltrafastSystems LLC, USA). The datasets were globally fitted with a kinetic model assuming a sequential population of excited states/species in cascade of nonreversible, decreasing rates (longer lifetimes). The fitting procedure of TA datasets gives EADS—evolution-associated difference spectra (van Stokkum et al. 2004). According to this model, the TA signal at any time delay and wavelength, Δ*A*(*t*,*λ*), can be reconstructed from superposition of *n*th *C*_*i*_(*t*) and EADS_*i*_(*λ*) products.

1 $$\Delta A(t,\lambda ) = \sum\limits_{i = 1}^{n} {C_{i} (t){\text{EADS}}_{i} (\lambda )}$$

*C*_*i*_(*t*) is the time-dependent concentration of *i*th *EADS* which is expressed as

2 $$\frac{{dC_{i} (t)}}{dt} = k_{i - 1} C_{i - 1} (t) - k_{i} C_{i} (t),i \ne 1,k_{i - 1} > k_{i}$$

and *C*_1_(*t*) is the populated by the excitation pulse that in spectrometer is represented as instrument response function, IRF:

3 $$\frac{{dC_{1} (t)}}{dt} = IRF(t) - k_{1} C_{1} (t)$$

The IRF was simulated by a Gaussian with a full-width at half-maximum (FWHM) of ~ 200 fs. Global analysis was performed using CarpetView 1.0 (Light Conversion Ltd., Lithuania). All plots were done in Origin 2019 (OriginLab Corp., USA).

## Results

### Steady-state absorption and fluorescence

**Fig. 1:**
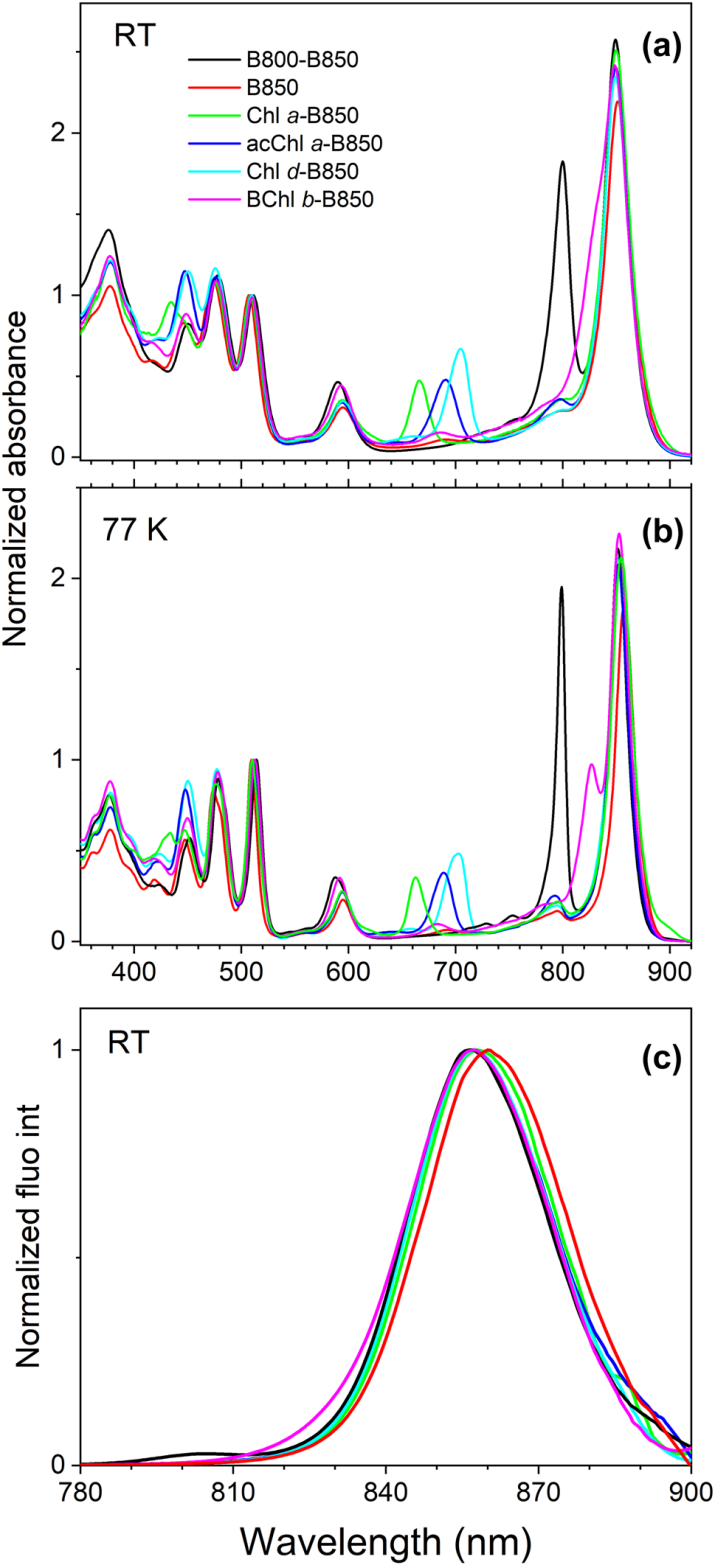
Absorption and fluorescence emission spectra of *Rba. sphaeroides* LH2 antenna complexes biding native and non-native (B)Chls in the B800 binding site. **a** RT and **b** 77 K absorption spectra of LH2 complexes: with a native BChl *a* in B800 binding site (B800–B850), B800-free (B850) and with the B800 site reconstituted with Chl *a*, acChl *a*, Chl *d*, and BChl *b*. **c** Normalized RT fluorescence emission spectra upon excitation at (0–0) vibronic band of Sphe (~ 510 nm)

Room temperature (RT) and 77 K absorption spectra (normalized at the Sphe (0–0) vibronic band at ~ 510 nm), and RT fluorescence emission spectra (normalized at their maxima) of the LH2 complexes are shown in Fig. 1. All LH2 complexes in this study contain the same Sphe carotenoid and native BChl *a* at the B850 site, the amplitudes of which are expected to remain consistent for all samples.

The B800–B850 LH2 is used as the benchmark, naturally binding BChl *a* in the B800 site with an absorption maximum at 800 nm. In this sample, the B850 band is positioned at 849 nm and B800/B850 peak ratio is 0.72. Typically, for in-tact LH2 this ratio ranges between 0.7 and 0.8 at room temperature and variation depends on purification protocols and storage buffers (Cong et al. 2008; Dilbeck et al. 2016). The (0–0) vibronic band of Sphe absorption appears at 512 nm. Removal of BChl *a* from the B800 binding sites (B850) only marginally alters spectroscopic features associated with other pigments. The B850 band is slightly bathochromically shifted to 851 nm and Sphe absorption is hypsochromically shifted with the (0–0) vibronic band appearing at 507 nm, in agreement with a previous study on the same LH2 complex (Bandilla et al. 1998; Clayton and Clayton 1981; Polivka et al. 2007). These spectral shifts are the result of structural rearrangements and loss of interactions with the B800 BChl *a*, altering the microenvironments of the B850 BChl *a* and Sphe pigments (Kramer et al. 1984; Robert and Frank 1988; Saga et al. 2017). Incorporation of non-native (B)Chls (Chl *a*, Chl *d*, acChl *a* and BChl *b*) into B800 binding site has no meaningful effect on the positions of Sphe and B850 absorption bands which appear within a 2-nm range with respect to native B800-B850 LH2. The *Q*_*y*_ band of the reconstituted (B)Chls appears at: 667 nm (Chl *a*), 691 nm (acChl *a*), 705 nm (Chl *d*), and ~ 830 nm (BChl *b*). Cryogenic temperatures (Fig. 1b) significantly improve the vibronic resolution of the absorption bands of Sphe and causes hypsochromic shifting of the *Q*_*y*_ band of B800 (B)Chls to up to 5 nm. The *Q*_*y*_ absorption band of BChl *b* is also clearly resolved at 77 K. Detailed information on the position of all spectral futures of these LH2 complexes at both RT and 77 K is listed in Table 1.

**Table 1:** Positions of spectral features in RT and 77 K absorption spectra of LH2 complexes consisting various (B)Chls at the B800 site

Complex	Spectral feature				
	BChl *a* Soret (nm)	Sphe S_0_ → S_2_ (0–0) (nm)	BChl *a Q*_*x*_ (nm)	B800 (nm)	B850 (nm)
RT					
B800–B850	376	511.5	590.5	800	849
B850	377.5	507.2	593	–	852
Chl *a*-B850	377.5	508.6	593	667	849
acChl *a*-B850	377.5	509.9	593	691	849
Chl *d*-B850	377.5	509.9	593	705	849
BChl *b*-B850	377.5	510.2	593	~ 830	849
77 K					
B800–B850	378	514.2	588	799	852
B850	378	509.7	595	–	858
Chl *a*-B850	378	510.4	595	663	854
acChl *a*-B850	378	511.5	595	689	854
Chl *d*-B850	378	512	595	703	854
BChl *b*-B850	378	512.7	592	827	853

Fluorescence emission spectra (normalized at their maxima) of all LH2 complexes recorded at RT are provided in Fig. 1c. Fluorescence emission peaks were at 857 nm for all except B850-LH2, which was at 860 nm. The weak emission band visible at 805 nm in the spectrum of B800–B850 LH2 (805/857 ratio of 0.03) is associated with direct fluorescence emission from B800 BChl *a*. Similarly, the expansion of the emission band between 810 and 840 nm in the spectrum of BChl *b*-B850 LH2 is associated with direct fluorescence emission from BChl *b*. Analogous, weak emission bands were recorded also for Chl-containing LH2s but are outside of the shown scale and were omitted for clarity.

**Fig. 2:**
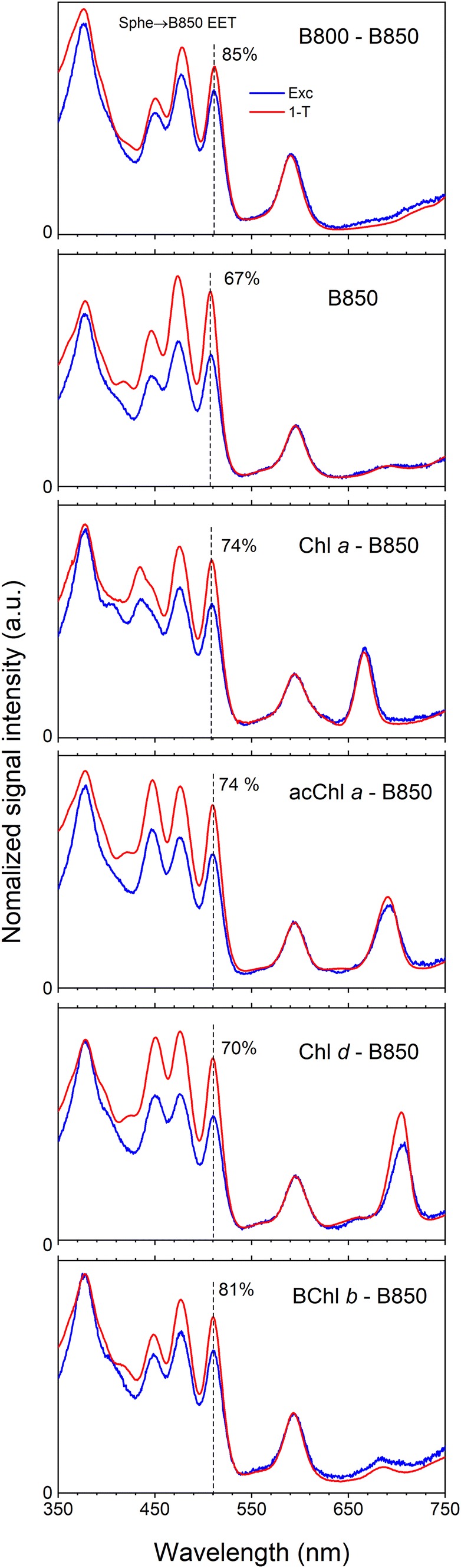
Fluorescence excitation spectra (Exc) and absorptance (1-transmittance (1-*T*)) spectra of *Rba. sphaeroides* LH2 complexes biding native and non-native (B)Chls in the B800 binding site recorded at RT. Fluorescence excitation profiles were recorded monitoring emission at 860 nm from B850 band. The spectral profiles are normalized to the same value at the *Q*_*x*_ band of BChl *a* (590 nm) assuming 100% EET to B850 *Q*_*y*_. Sphe-to-B850 EET was calculated at the indicated wavelength (vertical dashed line)

Fluorescence excitation spectra (monitoring emission from B850 BChl *a* at 860 nm) overlaid with absorptance (1-transmittance (1-*T*)) spectra are shown in Fig. 2. Energy transfer between non-native (B)Chls and B850 BChl *a* was extensively elaborated in past study (Swainsbury et al. 2019) and will not be discussed here. The main focus of this investigation is to determine how the overall Sphe-to-B850 BChl *a* energy transfer is affected by incorporating alternative (B)Chls into the B800 site. These pigments serve the role of intermediary in energy transfer between Sphe and the B850 BChl *a*, as has been well established for the native B800 BChl *a* (Macpherson et al. 2001; Polivka et al. 2007). All spectra were normalized at maximum of *Q*_*x*_ band of BChl *a* at 590 nm, assuming 100% energy transfer efficiency (EET) to B850 *Q*_*y*_ providing an internal standard to which the Sphe signals can be compared. In the benchmark B800-B850 LH2 complex, Sphe-to-B850 BChl *a* (Sphe → B850) EET was 85%, comparable to results from previous studies (Cong et al. 2008; Dilbeck et al. 2016; Rondonuwu et al. 2004). Removal of BChl *a* from B800 site (B850) causes a substantial reduction of the Sphe → B850 EET to 67%, clearly demonstrating that a substantial portion of excitation energy from the carotenoid is intermediately routed via B800 in agreement with previous studies (Macpherson et al. 2001; Polivka et al. 2007). Incorporation of BChl *b* into the B800 site restores Sphe → B850 EET to range comparable with the benchmark sample. Recovery of Sphe → B850 EET is smaller if Chls are reconstituted into B800 site, for Chl *d*, Sphe → B850 EET increases only marginally to 70% and to 74% for (ac)Chl *a*. Due to a substantial energy gap between the hypothetical fluorescence emission from the S_2_ electronic state of Sphe (hypothetically a mirror image of the absorption around the (0–0) vibronic band) and absorption spectrum of *Q*_*y*_ band of reconstituted (B)Chls, Sphe S_2_ → (B)Chls Soret → B850 excitation energy transfer is negligible. It is more complicated for BChl *a* and *b* because those pigments have strong absorption associated with the *Q*_*x*_ band at ~ 600 nm that substantially overlaps the theoretical S_2_ emission spectrum. This spectral overlap means that the Sphe S_2_ → BChls *Q*_*x*_ → B850 channel is also viable. However, for Chls with negligible strength of the *Q*_*x*_ transition, the Sphe S_1_ → Chls *Q*_*y*_ → B850 route will provide most of the extra contribution to the overall Sphe → B850 EET.

As mentioned in the introduction, the energy of the first excited singlet state (S_1_) of Sphe has previously been measured via various spectroscopic methods. Fluorescence emission spectroscopy revealed an extremely small quantum yield of fluorescence from the S_1_ state of 10^−7^ and a state energy of 14,200 cm^−1^ (704 nm) (Fujii et al. 1998), which was later supported by resonance Raman scattering (Sashima et al. 1998). On the other hand, transient absorption studies probing the S_1_ → S_2_ transition of Sphe in solvents placed the S_1_ state energy at 13,400 cm^−1^ (746 nm) (Niedzwiedzki et al. 2009; Polivka et al. 2001) and similar S_1_ state energy was proposed for Sphe embedded in LH2 based on results from TA (Polivka et al. 2002). Having in hand a set of LH2 complexes substituted with various (B)Chls in the B800 site allowed us to probe Sphe S_1_ state energy with TA using a very different approach. Steady-state absorption spectra of the LH2 complexes demonstrate that the *Q*_*y*_ band of reconstituted (B)Chls spans the 666–830 nm range and partially overlaps with the proposed 704–746 nm range of the Sphe S_1_ state. Therefore, one might expect that at some point both energies may coincide. When the Sphe S_1_ → (B)Chls *Q*_*y*_ excitation transfer route becomes permissible, it will manifest as a decrease in lifetime of the Sphe S_1_ excited state, which was examined by TA upon excitation of the Sphe (0–0) vibronic band.

### Transient absorption

**Fig. 3:**
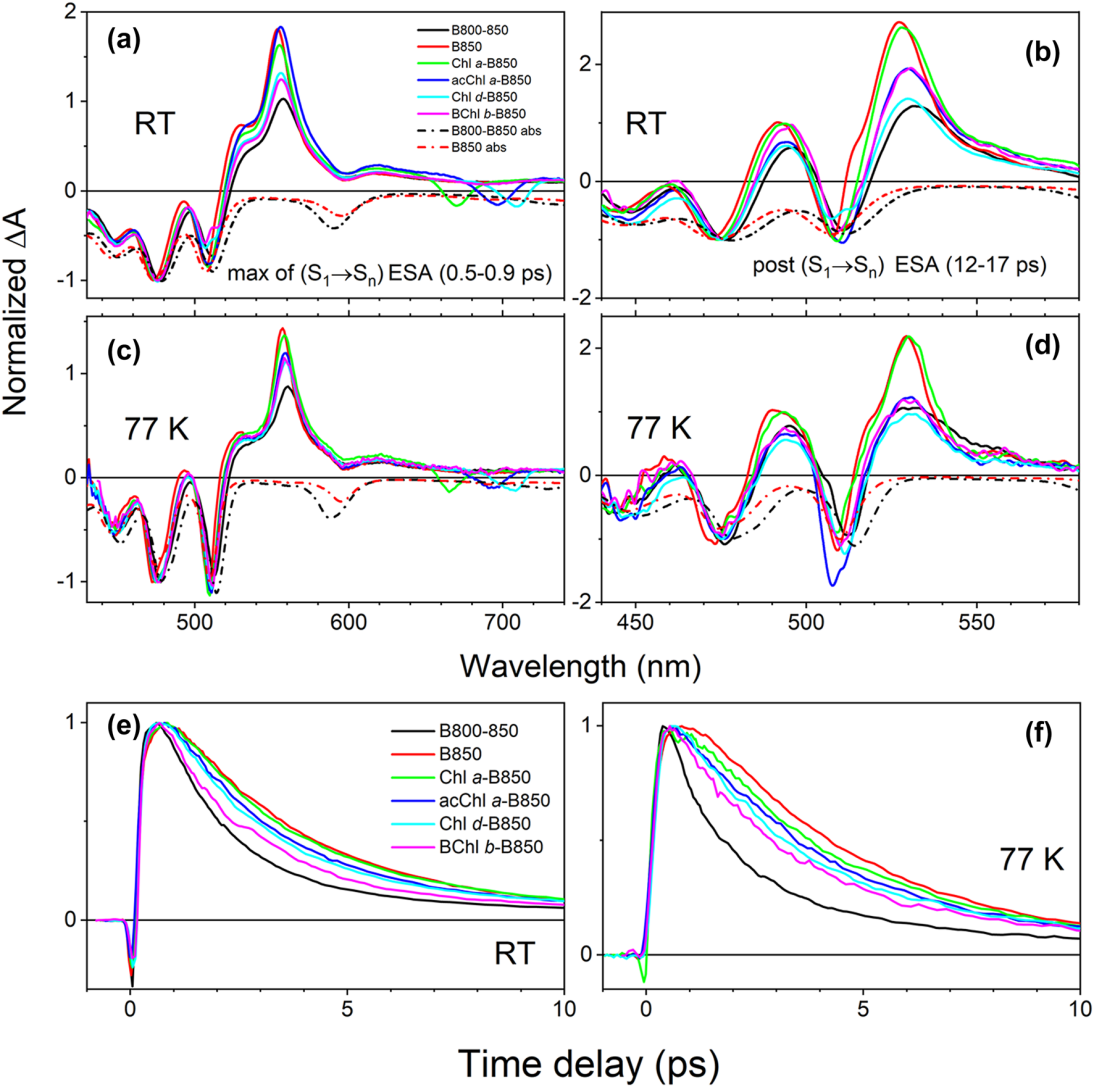
Transient absorption spectra and dynamics of Sphe S_1_ state in LH2 complexes with various (B)Chls in the B800 site. **a**, **b** RT and **c**, **d** 77 K TA spectra of Sphe recorded at time delays corresponding to maximum of S_1_ → S_n_ ESA (0.5–0.9 ps, **a**, **c**) and at delay times immediately after complete decay of the S_1_ state (12–17 ps, **b**, **d**). For better comparability, all TA spectra were normalized at Sphe (0–1) vibronic band. Inverted absorption spectra of B800–B850 and B850 LH2 are also shown. **e**, **f** Sphe S_1_ state dynamics probed at maximum of S_1_ → S_n_ ESA. The traces were recorded at RT and 77 K and are normalized at their maxima

Transient absorption measurements were taken at RT and 77 K. Figure 3 shows TA spectra of Sphe recorded at time delays corresponding to maximum signal of S_1_ → S_n_ excited state absorption (ESA) (0.5–0.9 ps) (Fig. 3a) and at delay times after complete decay of S_1_ state (12–17 ps) (Fig. 3b). For better comparability, all TA spectra were normalized at the (0-1) vibronic band of Sphe (~ 477 nm). To aid visualization, inverted steady-state absorption spectra of the B800-B850 and B850 LH2 are also provided (dashed lines) allowing better comparison of bleaching bands in the TA spectra with the shapes and positions of the Sphe vibronic bands. The TA spectra comprise several features, some of which are not associated with the carotenoid and correspond to bleaching of *Q*_*x*_ band of BChl *a* and *b*, and bleaching of *Q*_*y*_ bands of the reconstituted Chls. The bleaching of *Q*_*x*_ for B850 BChl *a* (595 nm—RT, 597 nm—77 K) and *Q*_*y*_ of Chl *a* (670 nm—RT, 665 nm—77 K), acChl *a* (696 nm—RT, 693 nm—77 K) and Chl *d* (709 nm—RT, 705 nm—77 K) were visible in the data. Bleaching of the *Q*_*x*_ band of BChl *b* is not clearly noticeable. It should be noted that bleaching of the Chl *Q*_*y*_ bands may not correspond well with their position in the steady-state absorption spectrum due to the overlapping contribution of a typically red-shifted stimulated emission.

The main transient feature associated with the carotenoid is the S_1_ → S_n_ ESA band that peaks at ~ 556 nm. Note that after normalization of the spectra to the ground state bleaching at 477 nm, the amplitude of the S_1_ → S_n_ ESA of Sphe differs for LH2s containing different (B)Chls in the B800 site. The bleaching of the Sphe ground state absorption should be the same in all cases because it corresponds to the initial pool of excited carotenoids. However, the amplitude of S_1_ → S_n_ ESA may differ in each LH2 as it will depend on what fraction of excited molecules (S_2_ state) will internally convert to the S_1_ state. There are various channels competing with S_2_ → S_1_ internal conversion (IC) including possible energy transfer to the *Q*_*y*_ bands of the (B)Chls directly or via *Q*_*x*_, or S_2_ mediated singlet-to-triplet fission. Given that the (B)Chls in the B800 site differently participate in these processes, there is no guarantee that in all LH2 complexes the same portion of excited carotenoids will internally convert to the S_1_ state. Data show that the decay of the spectral feature at ~ 530 nm takes substantially longer than that of S_1_ → S_n_ ESA, and that its true spectral shape became apparent at delay times after S_1_ → S_n_ ESA had completely decayed. Those spectra are shown in Fig. 3b and d. This spectral feature was previously observed and abbreviated as the S* transient spectrum, where S* corresponds to some vague excited singlet state (Cong et al. 2008; Papagiannakis et al. 2002, 2006). However, our recent TA investigations on various carotenoids in LH2 and LH1 demonstrated that S* may have different origins depending on the complex. In native B800-B850 LH2 S* could be explained as a combination of ground state bleaching of carotenoid within a Car^·+^-B800 BChl *a*^−^ pair, which forms with a reasonable yield upon direct excitation of carotenoid (Cong et al. 2008; Niedzwiedzki et al. 2016a), and a temporary electrochromic response of neutral carotenoid in proximity to a Car^·+^-B800 BChl *a*^−^ pair. In LH1 the S* feature is associated with quickly decaying carotenoid triplets populated via S_2_ singlet fission as it is reminiscent of *T*_1_ → *T*_*n*_ ESA (Kakitani et al. 2007; Niedzwiedzki et al. 2017). Therefore, the broad and flat positive band in the spectrum of the B800–B850 LH2 suggests that its TA spectrum is dominated by temporal electroabsorption of neutral Sphe, combined with bleaching of ground state absorption of Sphe involved in Sphe^·+^-B800 BChl *a*^−^ pair as was previously demonstrated for LH2 containing the carotenoid neurosporene (Niedzwiedzki et al. 2016a). In the B850 and Chl *a*-B850 LH2, the sharp positive band at 527 nm (RT) and 529 nm (77 K), which dominated TA spectra after decay of the S_1_ state of Sphe, suggests that the TA signals are mostly contributed by *T*_1_ → *T*_*n*_ ESA for Sphe bound to LH2 (Kakitani et al. 2007). Figure 3e and f shows Sphe S_1_ state dynamics (rise and decay) at RT and at 77 K in all studied LH2 systems. The dynamics were recorded at maximum of S_1_ → S_n_ ESA band and for better comparability are normalized at the maximum. Differences in Sphe S_1_ excited state lifetime upon incorporation of various (B)Chls into the B800 site were apparent. In order to get more precise information on dynamics of Sphe S_1_ state, kinetic traces from Fig. 3e and f were subjected to fitting with sum of exponentially decaying components convoluted by the IRF function of the spectrometer. Additionally, the TA datasets were also independently subjected to global fitting.

### Global fitting

**Fig. 4:**
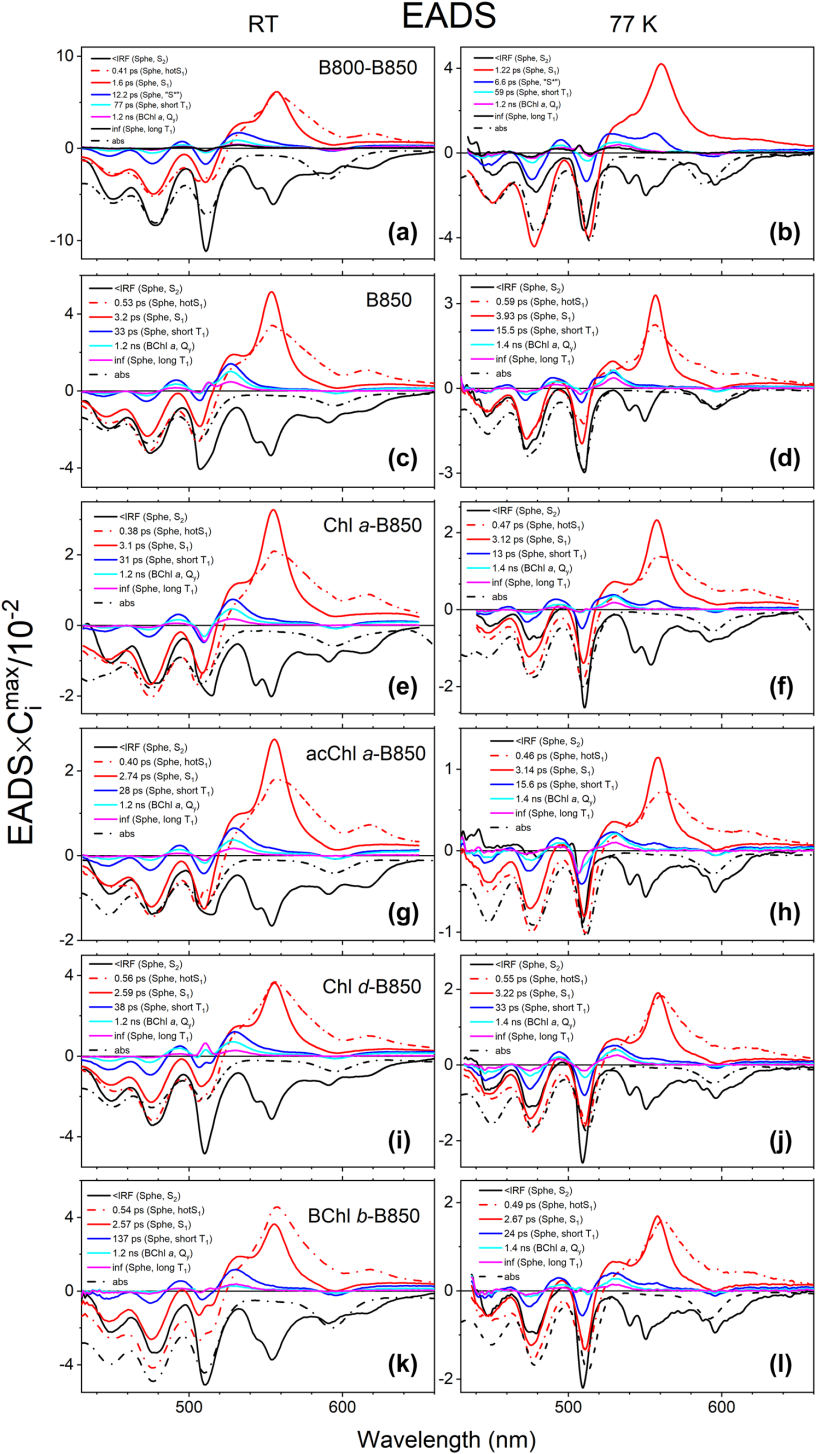
Global fitting results (EADS) of TA datasets obtained after excitation at (0–0) vibronic band of Sphe. The EADS were multiplied by maximal value of their time-dependent concentration (*C*_*i*_) and they represent relative contributions in the TA datasets. After the lifetime value of each EADS, a short description of the process associated with it is provided. For comparative purposes an inverted absorption spectrum of each LH2 complex is also shown. EADS—evolution-associated difference spectra, < IRF—lifetime shorter than FWHM of IRF function

The results of global fitting are shown in Fig. 4. The data were fitted according to unidirectional sequential model assuming irreversible and slower rates in each subsequent decay step. This kind of fitting of TA data gives EADS—evolution-associated difference spectra (van Stokkum et al. 2004). These are given for each LH2 complex at both RT and 77 K. The EADS were normalized to the amplitudes of their time-dependent concentrations *C*(*t*) to represent their contributions to the TA spectra. For reference, each panel also includes an inverted absorption spectrum of the LH2 that was adjusted to closely match bleaching of the Sphe absorption band. Each EDAS is color coded and their lifetimes are provided in the panel legend. In addition, legends contain short descriptions for each EADS. If the EADS lifetime was shorter than FWHM of the IRF (~200 fs) of the TA spectrometer, the lifetime was denoted as < IRF. It should be noted that EADS do not necessary represent the true transient spectra of a particular molecular species (e.g., specific excited state of Sphe or (B)Chl). However, if EADS are spectrally and temporally distinct their interpretation and assignment is rather straightforward. The first EADS (black solid line, present in all LH2s) is associated with decay of Sphe S_2_ excited state, known to have lifetime in the range of 150–250 fs depending on polarizability of the surrounding medium (Ricci et al. 1996). This EADS also has a clear S_2_ manifestation—bleaching of the state absorption and adjacent stimulated emission visible as negative bands at the long wavelength side of the absorption bleaching. The EADS with lifetime between 0.4 and 0.6 ps (dash-dot-dash, red), that is present in all fits except for 77 K B800–B850 LH2 is associated with vibrational relaxation of Sphe S_1_ (hotS_1_) excited electronic state. The EADS with characteristic sharp transient absorption band (S_1_ → S_n_ ESA band) at ~ 555 nm (solid red) is associated with decay of vibrationally equilibrated S_1_ state. Note that the S_1_ lifetime varies with (B)Chl incorporated to the B800 site from 1.6 ps (1.22 ps at 77 K) in the B800–B850 LH2 to 3.2 ps (3.93 ps at 77 K) in the B850 LH2 with various intermediate values for other (B)Chls. The EADS shown in solid blue with lifetime of ~ 12 ps (~ 6 ps at 77 K) for the B800–B850 was originally described as the “S*” feature associated with transient band of vague excited singlet state (Cong et al. 2008; Papagiannakis et al. 2002, 2006). As mentioned earlier, the spectral signature of this feature can be described as a combination electrochromic response of non-excited neutral carotenoid placed next to Sphe^·+^-B800 BChl *a*^−^ pair, always populated with significant yield in native LH2 complexes as previously demonstrated experimentally (Cong et al. 2008; Niedzwiedzki et al. 2016a; Polivka et al. 2004) and theoretically (Wormit and Dreuw 2006), and ground state bleaching of Sphe^·+^. This “S*” EADS is present only in global fitting results for the native B800–B850 LH2 as the presence of B800 BChl *a* is necessary for formation of the cation–anion pair, which appears to be unable to form with alternative B800 pigments. The blue color-coded EADS in the fitting results of the remaining LH2 complexes differs from the EADS present in fittings of the B800–B850 LH2. EADS associated with the electrochromic response have a much broader positive band while for all other “blue” EADS the bands are narrower, and these characteristics are enhanced at 77 K. Another EADS with lifetime of 77 ps has the same shape as EADS with an infinite lifetime; therefore, both are associated with transient band of the same excited state. Previously, these spectro-kinetic components were observed in neurosporene-containing LH2 with B800 BChl *a* removed and in LH1 complexes (Gradinaru et al. 2001; Niedzwiedzki et al. 2016a). These EADS are associated with fast and slow decay of the carotenoid triplet state, *T*_1_. It was demonstrated that in the absence of B800 BChl *a* in the antenna complex (B850 LH2 or any LH1) upon carotenoid direct excitation, *T*_1_ state very efficiently formed (Niedzwiedzki et al. 2016a, 2017) most likely via singlet fission (Tavan and Schulten 1987), presumably while carotenoid molecule is in the excited S_2_ state. This process apparently occurs for Sphe in all LH2 complexes reconstituted with non-native pigments in the B800 site—the S − *T* (singlet-minus-triplet) band of Sphe (ground state bleaching with adjunct *T*_1_ → *T*_*n*_ ESA band) is very evident. It is worth noticing that lifetime of the Sphe triplet formed via singlet fission was not uniform in all cases, which would suggest it is associated with triplet–triplet annihilation within the carotenoid and is not affected by (B)Chl reconstitution. In addition one would expect instantaneous triplet–triplet annihilation, which is linkly to occur within the temporal resolution of the spectrometer. Therefore, it is difficult to explain why the lifetime of the Sphe triplet pool varies substantially from complex to complex. Note that long-lived (essentially infinite in this time scale) Sphe triplets in the B800–B850 LH2 are formed via sensitization of the BChl *a* triplet and this process is slow and may not be as efficient as singlet fission. The cyan EADS with a lifetime of 1.2 ns (1.4 ns at 77 K) is associated with the recovery of excited B850 BChl *a* (B850*) and the major signature associated with this pigment is bleaching of the *Q*_*x*_ band at 600 nm. Note that in this case the EADS provides valuable information for the effective lifetimes of the molecular species that are formed during excitation decay; however, due to the complexity of the real pathway of excitation decay, their spectral shapes are only approximate and in some cases may be far away from real ones. This is due to mixing of simultaneously appearing/decaying signals. In order to visualize it, a more realistic, branching model that breaks the effective lifetimes into microscopic rates was also performed for the RT TA data of B800–B850 LH2 and is shown in Figure S1 for comparative purposes. A summary of the lifetimes for the excited Sphe S_1_ state are gathered in Table 2 along with results from single wavelength fits.

**Table 2:** Sphe excited S_1_ state dynamics obtained from global and single wavelength fittings

Complex	*τ*_hotS1_ (ps)	*τ*_S1_ (ps)	Fitting protocol	< *τ*_S1_ > ± SD^a^ (ps)
RT				
B800–B850	0.41	1.60	GF^c^	1.57 ± 0.04
	n.e.^b^	1.54	SW^d^ (557 nm)	
B850	0.53	3.20	GF	3.20 ± 0.00
	0.36	3.20	SW (555 nm)	
Chl *a*-B850	0.38	3.11	GF	3.00 ± 0.16
	0.36	2.88	SW (555 nm)	
acChl *a*-B850	0.40	2.74	GF	2.67 ± 0.10
	0.34	2.60	SW (556 nm)	
Chl *d*-B850	0.56	2.59	GF	2.51 ± 0.11
	n.e.	2.43	SW (556 nm)	
BChl *b*-B850	0.54	2.57	GF	2.40 ± 0.25
	n.e.	2.22	SW (557 nm)	
77 K				
B800–B850	n.e.	1.22	GF	1.22 ± 0.00
	n.e.	1.22	SW (559 nm)	
B850	0.59	3.93	GF	3.87 ± 0.09
	0.51	3.80	SW (557 nm)	
Chl *a*-B850	0.46	3.15	GF	3.29 ± 0.23
	n.e.	3.64	SW (558 nm)	
acChl *a*-B850	0.52	3.90	GF	3.40 ± 0.35
	0.45	3.51	SW (558 nm)	
Chl *d*-B850	0.55	3.22	GF	3.19 ± 0.05
	n.e.	3.15	SW (559 nm)	
BChl *b*-B850	0.49	2.67	GF	2.77 ± 0.14
	n.e.	2.87	SW (558 nm)	

^a^Averaged S_1_ state lifetime with standard deviation

^b^Not evident

^c^Global fitting

^d^Single wavelength fitting

## Discussion

### Estimate of S_1_ state energy for LH2-bound spheroidene

Global fitting of energy transfer from Sphe to substituted (B)Chls in the LH2 B800 site, particularly the trend in the lifetime of the Sphe S_1_ state, can be used to indirectly estimate the S_1_ state energy level of carotenoid bound within the LH2 complex. Currently for Sphe bound to LH2, only one experimental estimate of 13,400 cm^−1^ (746 nm) is available (Polivka et al. 2002). More classical spectroscopic measurements via recording fluorescence emission positioned the Sphe S_1_ state energy at 14,200 cm^−1^ (704 nm) in solvent (Fujii et al. 1998). Currently it is uncertain which value is more accurate as it is assumed that the solvent environment will not alter S_1_ state energy, meaning it may span the 13,400–14,200 cm^−1^ range. Our data show that the Sphe S_1_ → (B)Chls *Q*_*y*_ energy transfer rate depends upon the (B)Chl reconstructed in the B800 site; therefore, the energy of *Q*_*y*_ band, and by consequence its overlap with the hypothetical Sphe S_1_ state, influences the energy transfer process. This is an indication that energy transfer between Sphe and (B)Chls occurs via resonance transfer, characteristic for weakly coupled molecules (Scholes et al. 1997), which in principle requires good spectral overlap between emission of a donor (carotenoid) and absorption of an acceptor ((B)Chl *Q*_*y*_ band):

4 $$k_{\text{ET}} = \frac{1}{c\hbar }\left| T \right|^{2} J$$

5 $$J = \int\limits_{{ - \infty }}^{\infty } {F_{D} \left( \nu \right)\varepsilon _{A} \left( \nu \right)d\nu }$$

where *k*_ET_ is the rate constant for Sphe S_1_ → (B)Chl *Q*_*y*_ energy transfer, *T* is the electronic coupling term, *J* is the spectral overlap integral, *F*_D_ is a donor emission (S_1_, Sphe), and $\varepsilon_{\text{A}}$ is an acceptor absorption (*Q*_*y*_, (B)Chl).

**Fig. 5:**
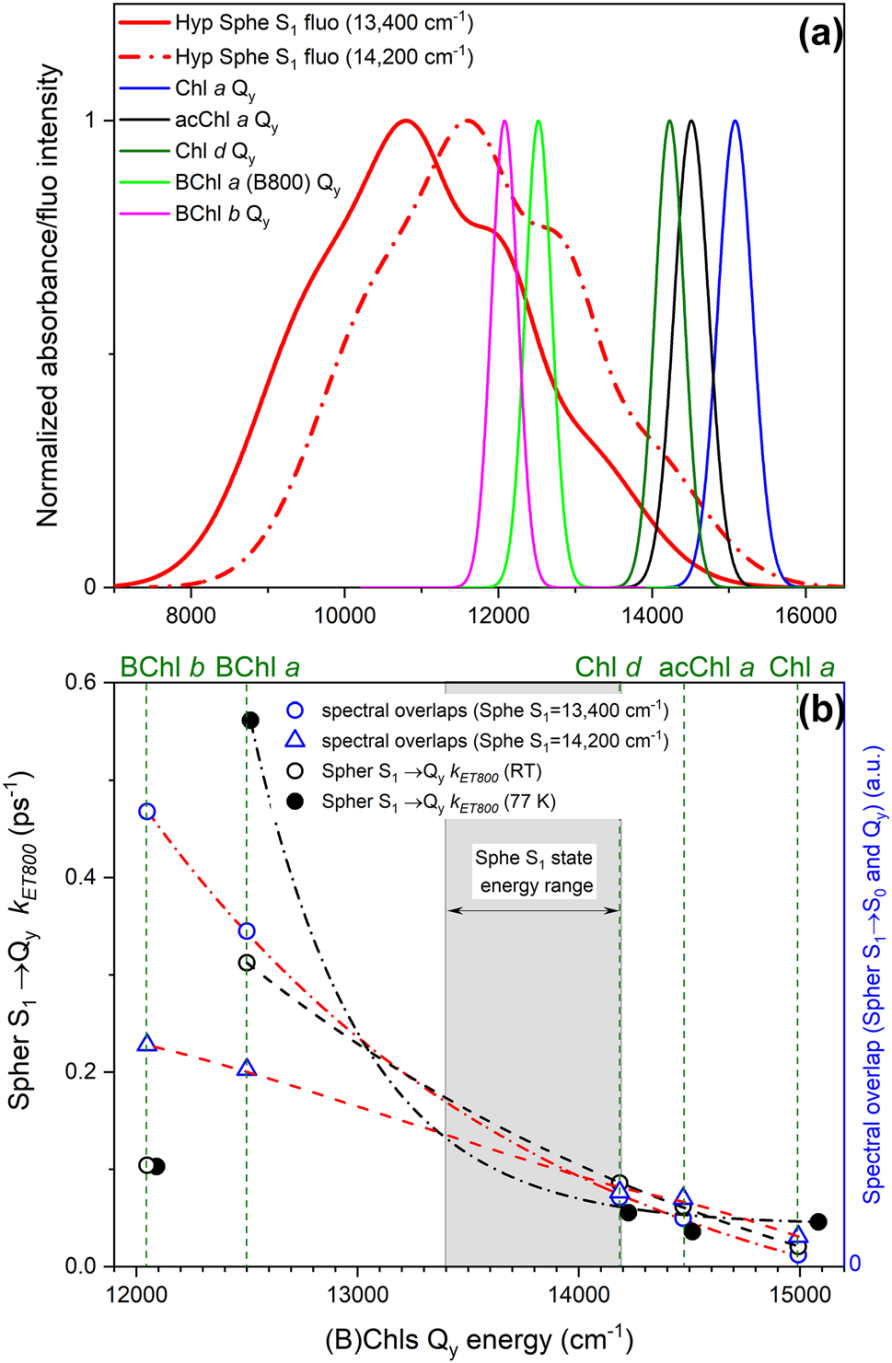
Spectral overlaps between hypothetical Sphe S_1_ fluorescence emission and (B)Chl *Q*_*y*_ band absorption and Sphe S_1_ → B800 (B)Chl *Q*_*y*_ rates (*k*_*ET800*_) as function of (B)Chl *Q*_*y*_ energy. (**a**) Hypothetical fluorescence emission spectrum of Sphe from the S_1_ state for two different energy levels (13,400 cm^−1^ and 14,200 cm^−1^) along with *Q*_*y*_ band absorption spectra of all (B)Chls present in studied LH2 complexes. These spectra were used to calculate spectral overlaps (*J*). (**b**) Experimental Sphe S_1_ → B800 (B)Chl *Q*_*y*_*k*_*ET*_ rates and calculated spectral overlaps (unitless, multiplied by scaling factor for the best coincidence) as a function of energy of *Q*_*y*_ band of (B)Chl present in the B800 site

Figure 5a shows spectra for the hypothetical emission from the S_1_ state of LH2-bound Sphe and *Q*_*y*_ absorption band of (B)Chls reconstructed to LH2, illustrating the overlap between them. Because we cannot acquire fluorescence emission spectra from the Sphe S_1_ state, it was mimicked by taking RT S_1_ emission of 3,4,5,6-tetrahydrospheroidene, a spheroidene analogue with shorter conjugation (*N* = 8), recorded in petroleum ether and shifting it to the expected S_1_ state energy of Sphe. The (0–0) vibronic band of the fluorescence spectrum was positioned at the hypothetical energies of the S_1_ state (13,400 cm^−1^—solid red, 14,200 cm^−1^—dash-dot red). The electronic coupling term (*T*) between Sphe and the B800-bound (B)Chl was assumed to be a constant as most of physical properties of Sphe and (B)Chl (distance, orientation, etc.) should remain similar. What clearly changes is the spectral overlap integral (*J*). The dependence of *J* on the energy/position of (B)Chl *Q*_*y*_ band, and its overlap with the Sphe S_1_ emission spectrum, is expected to relate directly to experimentally obtained Sphe S_1_ → (B)Chl *Q*_*y*_*k*_*ET*_ rates. The *k*_ET_ rates were obtained as follows. The effective/observed Sphe S_1_ state decay rate *k*_obs_ (*k*_obs_ = 1/*τ*_S1_) obtained from fitting of data from native and reconstituted LH2s consists of *k*_obs_ = *k*_int_ + *k*_ET850_ + *k*_ET800_, where *k*_int_ is intrinsic decay of the S_1_ state back to the ground state, and *k*_ET850_, *k*_ET800_ rates are associated with excitation energy transfer to both the B800 and B850 sites of LH2. For the LH2 without B800 (B850 LH2, rate denoted *k*′_obs_): *k*′_obs_ = *k*_int_ + *k*_ET850_. Clearly, it follows that the *k*_ET800_ rate can be obtained as: *k*_ET800_ =* k*_obs _− *k*′_obs_. The Sphe S_1_ → (B)Chl *Q*_*y*_*k*_ET_ rates obtained from fitting results of TA datasets are plotted in Fig. 5b in black symbols (open circles, RT, solid circles, 77 K). On the same graph, the spectral overlaps for both Sphe S_1_ energies are also plotted in blue symbols. As we do not have 77 K fluorescence emission of 3,4,5,6-tetrahydrospheroidene, spectral overlaps for cryogenic temperature were impossible to calculate. The overlaps have unitless values and the data were scaled to get the best coincidence with corresponding set of experimentally calculated *k*_ET800_ rates for comparative purposes. It is noticeable that *k*_ET800_ for Sphe S_1_ → BChl *b Q*_*y*_ is an apparent outlier from the overall trend. Our past studies demonstrated that this pigment correctly binds within the B800 site (Swainsbury et al. 2019) so this result is rather surprising. However, in one characteristic BChl *b* is unique with respect to all other (B)Chls. Once bound, its *Q*_*y*_ absorption band substantially overlaps (even at cryogenic temperature) with *Q*_*y*_ absorption band of the B850 BChl *a.* Consequently, B850 BChl *a *→ B800 BChl *b* excitation transfer is possible as it will be thermodynamically allowed. In this scenario, thermal equilibration of excited B850 BChl *a* and B800 BChl *b* will occur and the system will collectively decay with a common rate. This scheme has consequences for observed lifetime (*k*_ob_)^−1^ of the Sphe S_1_ state in BChl *b* LH2; statistically, upon excitation of the Sphe S_2_ state a substantial pool of excitations will be transferred to B850 BChl *a* by the S_2_ → *Q*_*x*_ route. After instantaneous IC, B850 BChl *a *→ B800 BChl *b* feedback could populate BChl *b Q*_*y*_ before it can accept excitation from the Sphe S_1_ state. Additionally, for B800 BChl *b*, Sphe S_2_ → BChl *b Q*_*x*_ excitation energy transfer is possible, which quickly populates BChl *b Q*_*y*_ via IC. With a few possible excitation transfer routes competing for the same population of molecules, the Sphe S_1_ → BChl *b Q*_*y*_ route is probably the most disadvantageous as it must first go through vibrational equilibration (as demonstrated in the fitting results) before transferring excitation energy. As the Sphe S_1_ → BChl *b Q*_*y*_ process may not be fast enough to compete with the other two processes, it is likely to be less efficient than one might expect; thus, BChl *b* was excluded from drawing conclusions on the overall trend. For the remaining (B)Chls, a remarkable correlation occurs between the *k*_ET800_ and spectral overlaps for Sphe S_1_ state energy at 13,400 cm^−1^. On the other hand, the trend in spectral overlaps for Sphe S_1_ state energy of 14,200 cm^−1^ is flatter and does not correlate well with the experimental *k*_ET800_. This is an indication that the previously suggested energy of 13,400 cm^−1^ is a good estimate of the actual value. This has additional confirmation in *k*_ET800_ plot at 77 K, which is still valuable despite our inability to compare it with spectral overlaps. It is expected that at 77 K vibronic bands of the fluorescence spectrum of Sphe will substantially narrow and spectral overlap will be more sensitive to position of the (B)Chl *Q*_y_ band. If the Sphe S_1_ state energy is indeed 14,200 cm^−1^ (704 nm) there should be a substantial difference in *k*_ET_ rates between Chl *a*, acChl *a* and Chl *d* because it is likely that only the *Q*_*y*_ band of Chl *d* (705 nm) will overlap the narrow emission from the Sphe (0–0) vibronic band. However, in the plot of experimental *k*_ET_ rates all three Chls are essentially the same, meaning the spectral overlap is similar for all of them. This suggests that the hypothetical Sphe fluorescence spectrum is shifted to lower energies (longer wavelengths) than the proposed 14,200 cm^−1^ level, again suggesting it resides closer to the 13,400 cm^−1^ level.

### In LH2 with non-native (B)Chls, the overall Sphe → B850 EET not clearly linked with the efficiency of the Sphe S_1_ → (B)Chl *Q*_*y*_ transfer route

The static fluorescence excitation experiments shown in Fig. 2 demonstrate that the trend in the Sphe → B850 EET does not follow with Sphe S_1_ → B800 (B)Chl *Q*_*y*_*k*_ET800_. This is due to several factors that may vary from complex to complex. One is the yield of singlet fission for Sphe in LH2s reconstructed with non-native (B)Chls. As this process practically eliminates substantial pool of excited carotenoids from participation in the energy transfer process (decreasing the overall Sphe → B850 EET), its presence is highly undesirable. It seems that the process is limited in the native B800-B850 LH2 but is always present at higher but different degree in LH2s with altered B800 sites. The second factor is less than 100% EET between reconstituted B800 (B)Chls and B850, as can be seen for Chl *d* in Fig. 2. Therefore, excitation energy transfer from the carotenoid to the intermediate B800 (B)Chl *a* may experience additional losses before it is accepted by B850. The last factor that should be considered is the Sphe S_2_ → B800 (B)Chl *Q*_*x*_ excitation energy transfer route. It seems that for BChls *a* and *b* in the B800 site this process may be considerably more efficient than the three Chls. This is because both BChls have a distinct *Q*_*x*_ band ~ 600 nm that allows energy transfer via S_2_ state while the Chls lack significant absorption in this spectral region.

## Summary

In this work we performed spectroscopic analysis of six variants of the LH2 antenna complex from *Rba. sphaeroides*, including native B800–B850, B800-free LH2 (B850) and four LH2 s with non-native (B)Chls reconstituted into the B800 site. We particularly focused how these modifications affect excitation energy transfer between naturally bound carotenoid Sphe and the B800 (B)Chls. Interestingly there is no apparent trend in the overall energy transfer from Sphe to B850 BChl *a*. This could be explained due to contributions of few, sometimes competing processes, that become active (or inactive) in LH2 s with altered B800 sites. On the other hand, the trend in the Sphe S_1_ → B800 (B)Chl *Q*_*y*_ energy transfer is noticeable and experimental outcomes could be used to check the validity of previously proposed energy values of the S_1_ state of Sphe embedded in LH2. The results support the LH2-bound Sphe S_1_ state energy in the vicinity of 13,400 cm^−1^ (746 nm).

## Electronic supplementary material

Below is the link to the electronic supplementary material.

Supplementary material 1 (DOCX 620 kb)

## Abbreviations

(B)Chl: (Bacterio)chlorophyll
EADS: Evolution-associated difference spectra
EET: Efficiency of excitation energy transfer
ESA: Excited state absorption
ET: Energy transfer
Exc: Excitation
FWHM: Full-width at half-maximum
IC: Internal conversion
LH1, 2: Light harvesting complex 1, 2
LDS: Lithium dodecyl sulfate
IRF: Instrument response function
RT: Room temperature
SD: Standard deviation
Sphe: Spheroidene
TA: Transient absorption
T-S: Triplet-minus-singlet

## References

[CR1] Angerhofer A, Bornhauser F, Gall A, Cogdell RJ (1995). Optical and optically detected magnetic-resonance investigation on purple photosynthetic bacterial antenna complexes. Chem Phys.

[CR2] Bandilla M, Ucker B, Ram M, Simonin I, Gelhaye E, McDermott G, Cogdell RJ, Scheer H (1998). Reconstitution of the B800 bacteriochlorophylls in the peripheral light harvesting complex B800-850 of *Rhodobacter sphaeroides* 2.4.1 with BChl a and modified (bacterio-)chlorophylls. Biochim Biophys Acta.

[CR3] Britton G, Liaaen-Jensen S, Pfander H, Britton G, Liaaen-Jensen S, Pfander H (1995). Carotenoids today and challenges for the future. Carotenoids: isolation and analysis, carotenoids.

[CR4] Britton G, Liaaen-Jensen S, Pfander H (2004). Carotenoids: handbook.

[CR5] Cartron ML, Olsen JD, Sener M, Jackson PJ, Brindley AA, Qian P, Dickman MJ, Leggett GJ, Schulten K, Hunter CN (2014). Integration of energy and electron transfer processes in the photosynthetic membrane of *Rhodobacter sphaeroides*. Biochim Biophys Acta.

[CR6] Chi SC, Mothersole DJ, Dilbeck P, Niedzwiedzki DM, Zhang H, Qian P, Vasilev C, Grayson KJ, Jackson PJ, Martin EC, Li Y, Holten D, Hunter CN (2014). Assembly of functional photosystem complexes in *Rhodobacter sphaeroides* incorporating carotenoids from the spirilloxanthin pathway. Biochim Biophys Acta.

[CR7] Christensen RL, Frank AJ, Young AJ, Britton D, Cogdell JW (1999). The electronic states of carotenoids. Photochemistry of carotenoids, advances in photosynthesis.

[CR8] Clayton RK, Clayton BJ (1981). B850 pigment-protein complex of *Rhodopseudomonas sphaeroides*—extinction coefficients, circular-dichroism, and the reversible binding of bacteriochlorophyll. Proc Natl Acad Sci USA.

[CR9] Cong H, Niedzwiedzki DM, Gibson GN, LaFountain AM, Kelsh RM, Gardiner AT, Cogdell RJ, Frank HA (2008). Ultrafast time-resolved carotenoid to-bacteriochlorophyll energy transfer in LH2 complexes from photosynthetic bacteria. J Phys Chem B.

[CR10] Demmig-Adams B, Adams WWI, Young AJ, Britton D (1993). The xanthophyll cycle. Carotenoids in photosynthesis.

[CR11] Dilbeck PL, Tang Q, Mothersole DJ, Martin EC, Hunter CN, Bocian DF, Holten D, Niedzwiedzki DM (2016). Quenching capabilities of long-chain carotenoids in light harvesting-2 complexes from *Rhodobacter sphaeroides* with an engineered carotenoid synthesis pathway. J Phys Chem B.

[CR12] Fidler AF, Singh VP, Long PD, Dahlberg PD, Engel GS (2013). Probing energy transfer events in the light harvesting complex 2 (LH2) of *Rhodobacter sphaeroides* with two-dimensional spectroscopy. J Chem Phys.

[CR13] Fiedor L, Heriyanto Fiedor J, Pilch M (2016). Effects of molecular symmetry on the electronic transitions in carotenoids. J Phys Chem Lett.

[CR14] Frank HA, Desamero RZB, Chynwat V, Gebhard R, vanderHoef I, Jansen FJ, Lugtenburg J, Gosztola D, Wasielewski MR (1997). Spectroscopic properties of spheroidene analogs having different extents of π-electron conjugation. J Phys Chem A.

[CR15] Frank HA, Bautista JA, Josue J, Pendon Z, Hiller RG, Sharples FP, Gosztola D, Wasielewski MR (2000). Effect of the solvent environment on the spectroscopic properties and dynamics of the lowest excited states of carotenoids. J Phys Chem B.

[CR16] Fraser NJ, Dominy PJ, Ucker B, Simonin I, Scheer H, Cogdell RJ (1999). Selective release, removal, and reconstitution of bacteriochlorophyll a molecules into the B800 sites of LH2 complexes from *Rhodopseudomonas acidophila* 10050. Biochemistry.

[CR17] Fujii R, Onaka K, Kuki M, Koyama Y, Watanabe Y (1998). The 2A_g_^−^ energies of all-*trans*-neurosporene and spheroidene as determined by fluorescence spectroscopy. Chem Phys Lett.

[CR18] Gradinaru CC, Kennis JT, Papagiannakis E, van Stokkum IH, Cogdell RJ, Fleming GR, Niederman RA, van Grondelle R (2001). An unusual pathway of excitation energy deactivation in carotenoids: singlet-to-triplet conversion on an ultrafast timescale in a photosynthetic antenna. Proc Natl Acad Sci USA.

[CR19] Greco JA, LaFountain AM, Kinashi N, Shinada T, Sakaguchi K, Katsumura S, Magdaong NC, Niedzwiedzki DM, Birge RR, Frank HA (2016). Spectroscopic investigation of the carotenoid deoxyperidinin: direct observation of the forbidden S_0_ → S_1_ transition. J Phys Chem B.

[CR20] Hashimoto H, Uragami C, Yukihira N, Gardiner AT, Cogdell RJ (2018). Understanding/unravelling carotenoid excited singlet states. J R Soc Interface.

[CR21] Herek JL, Fraser NJ, Pullerits T, Martinsson P, Polívka T, Scheer H, Cogdell RJ, Sundström V (2000). B800 → B850 energy transfer mechanism in bacterial LH2 complexes investigated by B800 pigment exchange. Biophys J.

[CR22] Hudson B, Kohler B (1974). Linear polyene electronic structure and spectroscopy. Annu Rev Phys Chem.

[CR23] Hunter CN, Turner G (1988). Transfer of genes-coding for apoproteins of reaction center and light-harvesting LH1 complexes to *Rhodobacter sphaeroides*. J Gen Microbiol.

[CR24] Jimenez R, Dikshit SN, Bradforth SE, Fleming GR (1996). Electronic excitation transfer in the LH2 complex of *Rhodobacter sphaeroides*. J Phys Chem.

[CR25] Kakitani Y, Akahane J, Ishii H, Sogabe H, Nagae H, Koyama Y (2007). Conjugation-length dependence of the T_1_ lifetimes of carotenoids free in solution and incorporated into the LH2, LH1, RC, and RC-LH1 complexes: possible mechanisms of triplet-energy dissipation. Biochemistry.

[CR26] Kereiche S, Bourinet L, Keegstra W, Arteni AA, Verbavatz JM, Boekema EJ, Robert B, Gall A (2008). The peripheral light-harvesting complexes from purple sulfur bacteria have different ‘ring’ sizes. FEBS Lett.

[CR27] Koepke J, Hu XC, Muenke C, Schulten K, Michel H (1996). The crystal structure of the light-harvesting complex II (B800-850) from *Rhodospirillum molischianum*. Structure.

[CR28] Kramer HJM, Vangrondelle R, Hunter CN, Westerhuis WHJ, Amesz J (1984). Pigment organization of the B800-850 antenna complex of *Rhodopseudomonas sphaeroides*. Biochem Biophys Acta.

[CR29] Linnanto J, Korppi-Tommola JEI (2002). Theoretical study of excitation transfer from modified B800 rings of the LH II antenna complex of *Rps. acidophila*. Phys Chem Chem Phys.

[CR30] Macpherson AN, Arellano JB, Fraser NJ, Cogdell RJ, Gillbro T (2001). Efficient energy transfer from the carotenoid S_2_ state in a photosynthetic light-harvesting complex. Biophys J.

[CR31] McDermott G, Prince SM, Freer AA, Hawthornthwaite-Lawless AM, Papiz MZ, Cogdell RJ, Isaacs NW (1995). Crystal structure of an integral membrane light-harvesting complex from photosynthetic bacteria. Nature.

[CR32] Niedzwiedzki DM, Blankenship RE (2018). Excited-state properties of the central-cis isomer of the carotenoid peridinin. Arch Biochem Biophys.

[CR33] Niedzwiedzki D, Koscielecki JF, Cong H, Sullivan JO, Gibson GN, Birge RR, Frank HA (2007). Ultrafast dynamics and excited state spectra of open-chain carotenoids at room and low temperatures. J Phys Chem B.

[CR34] Niedzwiedzki DM, Sandberg DJ, Cong H, Sandberg MN, Gibson GN, Birge RR, Frank HA (2009). Ultrafast time-resolved absorption spectroscopy of geometric isomers of carotenoids. Chem Phys.

[CR35] Niedzwiedzki DM, Kajikawa K, Aoki K, Katsumura S, Frank H (2013). Excited states energies and dynamics of peridinin analogues and the nature of the intramolecular charge transfer state in carbonyl-containing carotenoids. J Phys Chem B.

[CR36] Niedzwiedzki DM, Hunter CN, Blankenship RE (2016). Evaluating the nature of so-called S*-state feature in transient absorption of carotenoids in light-harvesting complex 2 (LH2) from purple photosynthetic bacteria. J Phys Chem B.

[CR37] Niedzwiedzki DM, Tronina T, Liu H, Staleva H, Komenda J, Sobotka R, Blankenship RE, Polivka T (2016). Carotenoid-induced non-photochemical quenching in the cyanobacterial chlorophyll synthase-HliC/D complex. Biochim Biophys Acta BBA.

[CR38] Niedzwiedzki DM, Swainsbury DJK, Martin EC, Hunter CN, Blankenship RE (2017). Origin of the S* excited state feature of carotenoids in light-harvesting complex 1 from purple photosynthetic bacteria. J Phys Chem B.

[CR39] Ostroumov EE, Mulvaney RM, Cogdell RJ, Scholes GD (2013). Broadband 2D electronic spectroscopy reveals a carotenoid dark state in purple bacteria. Science.

[CR40] Papagiannakis E, Kennis JT, van Stokkum IH, Cogdell RJ, van Grondelle R (2002). An alternative carotenoid-to-bacteriochlorophyll energy transfer pathway in photosynthetic light harvesting. Proc Natl Acad Sci USA.

[CR41] Papagiannakis E, van Stokkum IH, Vengris M, Cogdell RJ, van Grondelle R, Larsen DS (2006). Excited-state dynamics of carotenoids in light-harvesting complexes. 1. Exploring the relationship between the S_1_ and S* states. J Phys Chem B.

[CR42] Papiz MZ, Prince SM, Howard T, Cogdell RJ, Isaacs NW (2003). The structure and thermal motion of the B800-850 LH2 complex from *Rps. acidophila* at 2.0Å resolution and 100 K: new structural features and functionally relevant motions. J Mol Biol.

[CR43] Pendon ZD, Gibson GN, van der Hoef I, Lugtenburg J, Frank HA (2005). Effect of isomer geometry on the steady-state absorption spectra and femtosecond time-resolved dynamics of carotenoids. J Phys Chem B.

[CR44] Polivka T, Herek JL, Zigmantas D, Akerlund HE, Sundstrom V (1999). Direct observation of the (forbidden) S_1_ state in carotenoids. Proc Natl Acad Sci USA.

[CR45] Polivka T, Zigmantas D, Frank HA, Bautista JA, Herek JL, Koyama Y, Fujii R, Sundstrom V (2001). Near-infrared time-resolved study of the S_1_ state dynamics of the carotenoid spheroidene. J Phys Chem B.

[CR46] Polivka T, Zigmantas D, Herek JL, He Z, Pascher T, Pullerits T, Cogdell RJ, Frank HA, Sundstrom V (2002). The carotenoid S_1_ state in LH2 complexes from purple bacteria *Rhodobacter sphaeroides* and *Rhodopseudomonas acidophila*: s_1_ energies, dynamics, and carotenoid radical formation. J Phys Chem B.

[CR47] Polivka T, Pullerits T, Frank HA, Cogdell RJ, Sundstrom V (2004). Ultrafast formation of a carotenoid radical in LH2 antenna complexes of purple bacteria. J Phys Chem B.

[CR48] Polivka T, Niedzwiedzki D, Fuciman M, Sundstrom V, Frank HA (2007). Role of B800 in carotenoid-bacteriochlorophyll energy and electron transfer in LH2 complexes from the purple bacterium *Rhodobacter sphaeroides*. J Phys Chem B.

[CR49] Polivka T, Frank HA, Enriquez MM, Niedzwiedzki DM, Liaaen-Jensen S, Hemming J, Helliwell JR, Helliwell M (2010). X-ray crystal structure and time-resolved spectroscopy of the blue carotenoid violerythrin. J Phys Chem B.

[CR50] Prince SM, Papiz MZ, Freer AA, McDermott G, Hawthornthwaite-Lawless AM, Cogdell RJ, Isaacs NW (1997). Apoprotein structure in the LH2 complex from *Rhodopseudomonas acidophila* strain 10050: modular assembly and protein pigment interactions. J Mol Biol.

[CR51] Qian P, Papiz MZ, Jackson PJ, Brindley AA, Ng IW, Olsen JD, Dickman MJ, Bullough PA, Hunter CN (2013). Three-dimensional structure of the *Rhodobacter sphaeroides* RC-LH1-PufX complex: dimerization and quinone channels promoted by PufX. Biochemistry.

[CR52] Ricci M, Bradforth SE, Jimenez R, Fleming GR (1996). Internal conversion and energy transfer dynamics of spheroidene in solution and in the LH-1 and LH-2 light-harvesting complexes. Chem Phys Lett.

[CR53] Robert B, Frank HA (1988). A resonance Raman investigation of the effect of lithium dodecyl-sulfate on the B800-850 light-harvesting protein of *Rhodopseudomonas*-*acidophila*-7750. Biochem Biophys Acta.

[CR54] Rondonuwu FS, Watanabe Y, Fujii R, Koyama Y (2003). A first detection of singlet to triplet conversion from the 1^1^B_u_^−^ to the 1^3^A_g_ state and triplet internal conversion from the 1^3^A_g_ to the 1^3^B_u_ state in carotenoids: dependence on the conjugation length. Chem Phys Lett.

[CR55] Rondonuwu FS, Yokoyama K, Fujii R, Koyama Y, Cogdell RJ, Watanabe Y (2004). The role of the 1^1^B_u_^−^ state in carotenoid-to-bacteriochlorophyll singlet-energy transfer in the LH2 antenna complexes from *Rhodobacter sphaeroides* G1C, *Rhodobacter sphaeroides* 2.4.1, *Rhodospirillum molischianum* and *Rhodopseudomonas acidophila*. Chem Phys Lett.

[CR56] Saga Y, Hirota K, Asakawa H, Takao K, Fukuma T (2017). Reversible changes in the structural features of photosynthetic light-harvesting complex 2 by removal and reconstitution of B800 bacteriochlorophyll *a* pigments. Biochemistry.

[CR57] Sashima T, Shiba M, Hashimoto H, Nagae H, Koyama Y (1998). The 2A_g_^−^ energy of crystalline all-*trans*-spheroidene as determined by resonance-Raman excitation profiles. Chem Phys Lett.

[CR58] Schmidt M, Tavan P (2012). Electronic excitations in long polyenes revisited. J Chem Phys.

[CR59] Scholes GD, Harcourt RD, Fleming GR (1997). Electronic interactions in photosythetic light-harvesting complexes: the role of carotenoids. J Phys Chem B.

[CR60] Schulten K, Karplus M (1972). On the origin of a low-lying forbidden transition in polyenes and related molecules. Chem Phys Lett.

[CR61] Staleva H, Komenda J, Shukla MK, Slouf V, Kana R, Polivka T, Sobotka R (2015). Mechanism of photoprotection in the cyanobacterial ancestor of plant antenna proteins. Nat Chem Biol.

[CR62] Swainsbury DJK, Faries KM, Niedzwiedzki DM, Martin EC, Flinders AJ, Canniffe DP, Shen GZ, Bryant DA, Kirrnaier C, Holten D, Hunter CN (2019). Engineering of B800 bacteriochlorophyll binding site specificity in the *Rhodobacter sphaeroides* LH2 antenna. Biochim Biophys Acta.

[CR63] Takaichi S, Frank HA, Young AJ, Britton G, Cogdell JW (1999). Carotenoids and carotenogenesis in anoxygenic photosythetic bacteria. Photochemistry of carotenoids, advances in photosythesis.

[CR64] Tavan P, Schulten K (1987). Electronic excitations in finite and infinite polyenes. Phys Rev B.

[CR65] Trinkunas G, Herek JL, Polivka T, Sundstrom V, Pullerits T (2001). Exciton delocalization probed by excitation annihilation in the light-harvesting antenna LH2. Phys Rev Lett.

[CR66] van Stokkum IHM, Larsen DS, van Grondelle R (2004). Global and target analysis of time-resolved spectra. Biochim Biophys Acta BBA.

[CR67] Walz T, Jamieson SJ, Bowers CM, Bullough PA, Hunter CN (1998). Projection structures of three photosynthetic complexes from *Rhodobacter sphaeroides*: LH2 at 6 angstrom LH1 and RC-LH1 at 25 angstrom. J Mol Biol.

[CR68] Wormit M, Dreuw A (2006). Carotenoid radical cation formation in LH2 of purple bacteria: a quantum chemical study. Journal of Physical Chemistry B.

[CR69] Zhang JP, Fujii R, Qian P, Inaba T, Mizoguchi T, Onaka K, Watanabe Y, Nagae H, Koyama Y (2000). Mechanism of the carotenoid-to-bacteriochlorophyll energy transfer via the S_1_ state in the LH2 complexes from purple bacteria. Journal of Physical Chemistry B.

