# Supplementary material for: Carotenoid-to-(bacterio)chlorophyll energy transfer in LH2 antenna complexes from Rba. sphaeroides reconstituted with non-native (bacterio)chlorophylls
Source: Photosynth Res. 2019 Jul 26;144(2):155–69. doi: 10.1007/s11120-019-00661-6 (PMC7203092; doi:10.1007/s11120-019-00661-6)
Supplement: Supplementary file 1 — Supplementary material 1 (DOCX 620 kb) [file 11120_2019_661_MOESM1_ESM.docx]

Supplementary Information

for

**Carotenoid-to-(Bacterio)Chlorophyll Energy Transfer in LH2 Antenna Complexes from *Rba. sphaeroides* Reconstituted with Non-native (Bacterio)Chlorophylls**

Dariusz M. Niedzwiedzki^1,2,*^ David J.K. Swainsbury^3^ and C. Neil Hunter^3^

^1^Center for Solar Energy and Energy Storage and ^2^Department of Energy, Environmental & Chemical Engineering, Washington University, St. Louis, MO 63130 USA

^3^ Department of Molecular Biology and Biotechnology, University of Sheffield, Sheffield, S10 2TN, United Kingdom

*Corresponding Author: Dr. Dariusz M. Niedzwiedzki, Washington University, St. Louis, MO 63130 USA, [niedzwiedzki@wustl.edu](mailto:niedzwiedzki@wustl.edu)


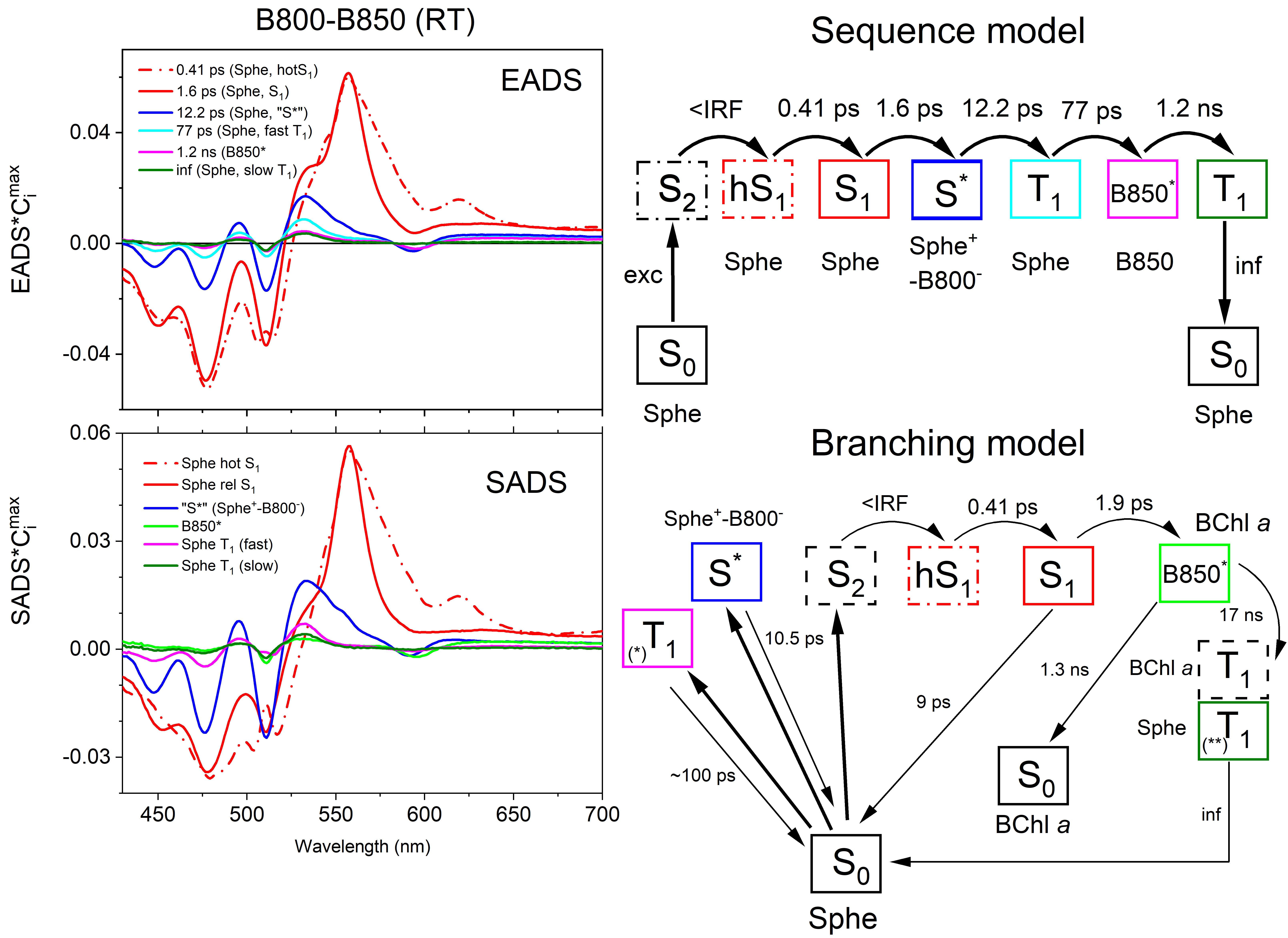


Figure S1: EADS vs SADS for B800-850 LH2 and associated fitting models that were used for data modeling. (*) – This spheroidene triplet pool is formed via singlet fission and represent “fast” decaying triplets from the graph. (**) - Quenching of the B850 BChl *a* triplets is essentially instantaneous and for fitting purpose it was assumed that Sphe T_1_ is populated directly from B850*. Sphe S_2_ SADS was removed for figure clarity; SADS- species associated difference spectra – amplitude spectra resulting from targeted modelling.

Note that improving of fitting model toward one that is more and more realistic leads to a better spectral separation (eliminates spectral missing due to temporal overlap) of the spectra associated with transient molecular species formed during excitation decay/migration process (e.g. compare shapes of the Sphe S_1_ SADS and EADS).
